# Methodology for the Determination of Fruit, Vegetable, Nut and Legume Points for Food Supplies without Quantitative Ingredient Declarations and Its Application to a Large Canadian Packaged Food and Beverage Database

**DOI:** 10.3390/foods9081127

**Published:** 2020-08-15

**Authors:** Laura Vergeer, Mavra Ahmed, Beatriz Franco-Arellano, Christine Mulligan, Kacie Dickinson, Jodi T. Bernstein, Marie-Ève Labonté, Mary R. L’Abbé

**Affiliations:** 1 Department of Nutritional Sciences, Faculty of Medicine, University of Toronto, Toronto, ON M5S 1A8, Canada; laura.vergeer@mail.utoronto.ca (L.V.); mavz.ahmed@utoronto.ca (M.A.); beatriz.francoarellano@mail.utoronto.ca (B.F.-A.); christine.mulligan@mail.utoronto.ca (C.M.); kacie.dickinson@flinders.edu.au (K.D.); jodi.bernstein@mail.utoronto.ca (J.T.B.); marie-eve.labonte@fsaa.ulaval.ca (M.-È.L.); 2 Caring Futures Institute, College of Nursing and Health Sciences, Flinders University, Adelaide, SA 5001, Australia; 3 Centre Nutrition, Santé et Société (NUTRISS), Institute of Nutrition and Functional Foods, Laval University, Québec City, QC G1V 0A6, Canada

**Keywords:** nutrient profiling, FVNL points, ingredients, nutrition policy

## Abstract

Nutrient profiling (NP) models are useful tools for objectively and transparently quantifying the nutritional quality of packaged foods and beverages. Many NP models incorporate ingredients beneficial for health (e.g., fruits, vegetables, nuts, legumes (FVNL)) in addition to less healthful nutrients or components, assigning points based on the proportion of the product that contains FVNL ingredients. However, with food labelling in most countries lacking mandatory quantitative ingredient declarations (QUIDs), there is potential for the estimation of FVNL points to be ambiguous and inconsistent. The purpose of this article was to describe the development and application of methodology for estimating FVNL points for products without QUIDs, based on the position of FVNL components within the ingredients list. Using this method, FVNL points were calculated for packaged foods and beverages in the University of Toronto Food Label Information Program 2017 database (*n* = 17,337). Distributions of FVNL points were examined overall and by food category. This study provides evidence of the feasibility of this method in distinguishing between products with differing amounts of FVNL ingredients. This method will be valuable for researchers and policymakers in ensuring consistent, objective and reproducible estimations of FVNL points—and consequently, assessments of product healthfulness—for food supplies without QUIDs.

## 1. Introduction

Nutrient profiling (NP) has been recognized by the World Health Organization (WHO), governments and other authoritative bodies as an objective, transparent and reproducible method for assessing the nutritional quality of packaged food and beverage products [1]. It involves classifying or ranking foods based on their nutritional composition for various public health nutrition policy purposes, with the ultimate aim of preventing diet-related chronic disease and promoting health. Although applications of NP date back as far as the 1980s [2,3], it has received greater attention since 2010 when the WHO recommended that its Member States use NP models to determine whether a product is sufficiently healthy to be marketed to children and adolescents under 18 years of age, and therefore restrict the marketing of less healthy foods and beverages [4]. In addition to regulations concerning unhealthy food marketing to children, NP is suitable for use in various other government and industry food environment policies, such as those concerning front-of-package (FOP) nutrition labelling, nutrition and health claims, food taxes, reformulation and school food programs, among others [1].

In recent years, a number of NP models have been introduced by governments or public health agencies to fulfill these different purposes [5]. While some NP models focus exclusively on nutrients recommended to limit in the diet (e.g., sodium, saturated fat, total or free sugars), others also incorporate nutrients or components to encourage, such as protein, fibre and/or fruits and vegetables. Several models account for the fruit, vegetable, nut and legume (FVNL) content of a product, including the Ofcom NP model [6], the Food Standards Australia New Zealand (FSANZ) Nutrient Profiling Scoring Criterion (NPSC) [7], the Health Star Rating (HSR) system [8], and the Nutri-Score (which also considers the content of rapeseed, walnut and olive oils as part of its positive nutrients and components) [9]. The Ofcom NP model was developed by the UK Food Standards Agency in 2004–2005 to regulate food advertising to children on television [6], while the latter three models are derivatives of the Ofcom model [7,8,9]. The FSANZ NPSC was designed to regulate the use of health claims on foods in Australia and New Zealand [10]. It was followed in 2014 by the introduction of the HSR system in Australia and New Zealand as a voluntary FOP labelling system to help consumers select healthier foods [11]. Nutri-Score is among the newest government-endorsed NP models and is currently being considered for implementation as a voluntary FOP labelling system by governments in France, Spain, Belgium, Germany, Switzerland, the Netherlands and Luxembourg [12,13]. With the increasingly widespread use of NP models to evaluate the healthfulness of packaged food supplies around the world, it will be crucial to maximize the accuracy and consistency of their application, including the calculation of points for FVNL, and rapeseed, walnut and olive oils under the Nutri-Score.

FVNL points are assigned to a product based on its ingredients list, which is examined to determine whether FVNL components constitute a significant proportion of the product by weight [7,8,9]. In several countries and regions (e.g., the European Union, the United Kingdom, Australia, New Zealand), most packaged foods and beverages are required to display quantitative ingredient declarations (QUIDs) expressed as percentages or weights [14,15,16]. While these regulations vary by country, QUIDs are typically required when the ingredient appears in the name of the food (e.g., strawberries in ‘strawberry’ yogurt), is otherwise emphasized on the product packaging (e.g., a picture of a strawberry on the yogurt container) and/or is commonly associated with the product by consumers (e.g., beans as a component of chili) [14,15,16]. Although QUIDs are considered a best practice in food labelling [17], they are currently not required in most countries, including Canada and the United States. Canadian regulations do, however, state that ingredients must be listed in descending order of their proportion by weight or percentage of a packaged product [18]. A cross-sectional analysis of the Canadian packaged food supply in 2013 found that while 100% of the sampled products displayed a list of ingredients, only 2.6% included QUIDs [19]. In the absence of QUIDs, assigning FVNL points to products can be challenging and somewhat ambiguous, with the potential for considerable inconsistency between studies.

The purpose of this study was to develop a method for the estimation of FVNL points for products in food supplies without QUIDs, using a set of criteria that can be easily applied by other researchers and to other food supplies lacking QUIDs. While older, abbreviated versions of these methods have been published [20,21], they include limited detail and do not account for recent important developments, including the introduction of newer NP models (e.g., the Nutri-Score, the HSR system) and modifications to pre-existing models. A secondary objective of this study was to use the method to examine the distribution of FVNL points within food categories, drawing on a large and highly representative sample of Canadian packaged food and beverage products. The latter objective may be useful for policymakers to understand the distribution of FVNL points and the potential impact of proposed changes to NP models such as the HSR system and the Ofcom model, which are currently under review (and will likely be revised in the near future) [22,23].

## 2. Materials and Methods 

### 2.1. Food Composition Data

This study involved a cross-sectional analysis of the University of Toronto Food Label Information Program (FLIP) 2017, which is described in detail elsewhere [24]. In summary, FLIP 2017 is a database of packaged food and beverage product labels that were collected from retail outlets of three major Canadian grocery chains (Loblaws, Metro and Sobeys) in Toronto, Canada between June and September of 2017. FLIP contains information such as: product name, brand and company; Nutrition Facts table (NFt); ingredients list; and photos of all sides of the product packaging. Products were assigned to one of the 24 major and 153 minor food categories described in Health Canada’s Table of Reference Amounts for Food (TRA) [25].

### 2.2. Differences in Assignation of FVNL Points According to Different NP Models

The procedure for calculating FVNL points (using QUIDs) is described in reports outlining the application of the Ofcom NP Model [6], the FSANZ NPSC [7], the HSR system [8] and the Nutri-Score [9]. While the basis of the point systems and the procedure for calculating them are similar between NP models, there are some key differences. For example, although all FVNL point scales are based on the percentage of the product that is FVNL, the range of possible points differs between NP models, with some FVNL point systems differentiating between concentrated and non-concentrated products (e.g., the Ofcom model, the FSANZ NPSC and the HSR system), or between foods and beverages (i.e., the Nutri-Score) (Table 1). The eligibility of certain ingredients for FVNL points also differs slightly between NP models. Notably, the Nutri-Score is the only NP model to account for rapeseed, walnut and olive oils to align with European dietary recommendations concerning oils [9]. Additionally, tubers (i.e., potatoes and other starchy vegetables) are counted as FVNL under the FSANZ NPSC and HSR system, but not for the Nutri-Score or Ofcom NP models [6,7,8,9]. Other foods that are eligible for FVNL points under the FSANZ NPSC and HSR system, but not the Nutri-Score, include capers, spices, pine nuts, and chia, poppy, sunflower or flax seeds. 

### 2.3. Description of Research Team’s Methodology for Estimating FVNL Points

Since 2015, members of our research team have been developing methods for assigning FVNL points to products in the FLIP database based on the position of FVNL ingredients within the ingredients list. Development of these criteria was iterative, with revisions made as updates were made to relevant NP models and as new types of products appeared on the market and in the FLIP database. When making key decisions regarding the application of FVNL points, the research team also consulted with academic experts applying these NP models in other countries.

**Figure 1:**
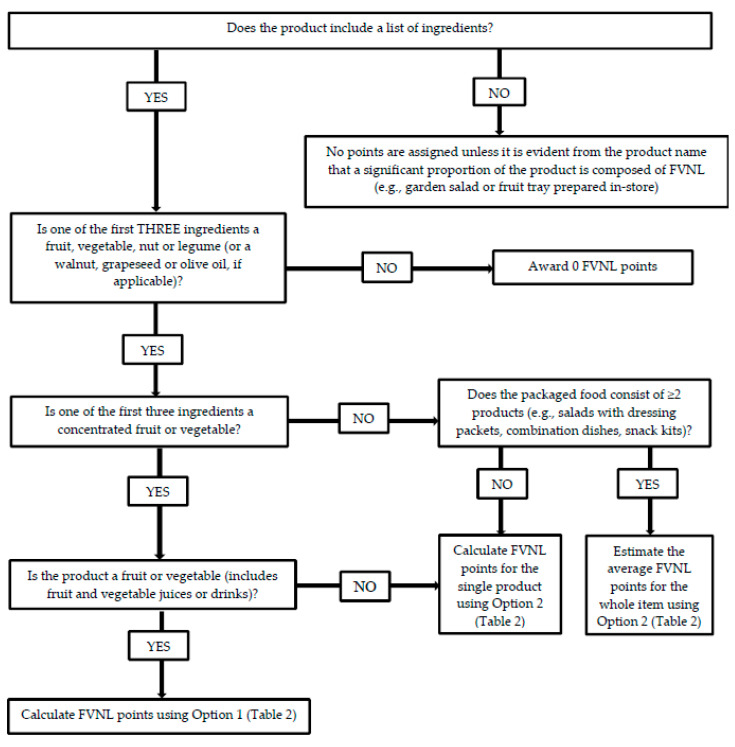
Decision tree for estimating fruit, vegetable, nut and legume (FVNL) points for products without quantitative ingredient declarations for the purposes of applying the Ofcom Nutrient Profile Model, the Food Standards Australia New Zealand Nutrient Profiling Scoring Criterion, the Health Star Rating system, and the Nutri-Score.

The research team’s decision tree is outlined in Figure 1 and the criteria for assigning FVNL points are described in Table 2. Consistent with the guidelines for applying the Ofcom NP model, the FSANZ NPSC and the HSR system, separate criteria were established for concentrated (“Option 1”) and non-concentrated (“Option 2”) products (Table 2), using the FVNL point scales shown in Table 1. Under Option 1, for a product to score at least 1 point, a concentrated FVNL component must be one of the first three ingredients listed. Using the order of the ingredients list, the fourth ingredient could account for, at most, one quarter (25%) of a product’s weight, which would occur if there were only four ingredients that all appeared in equal amounts. Considering this, it is likely that the fourth ingredient has a percentage weight below the 25% threshold required to score 1 point. Under Option 2, for a product to score at least 1 point, a FVNL must be one of the first two ingredients listed. Using the order of the ingredients list, the third ingredient could account for, at most, one third (33%) of a product’s weight; 33% is therefore below the threshold of > 40% required to score 1 point.

To estimate the FVNL points for a product, it is first determined whether the product includes a list of ingredients (Figure 1). Although most packaged foods and beverages in Canada must display an ingredients list, certain types of products can be exempt from this requirement; for example, ready-meals or baked goods prepared in-store. For products without an ingredients list, no FVNL points are assigned unless it is evident from the name of the product that it constitutes a significant proportion of FVNL (e.g., a garden salad, or a fruit or vegetable tray prepared in-store). If the product contains an ingredients list, the first three ingredients are examined to determine whether one or more of them are FVNL. If FVNL components are identified as one of the first three ingredients, it is then determined whether one or more of these ingredients are concentrated. If the product is a fruit or vegetable (including fruit and vegetable juices or drinks) containing FVNL components that are all (or mainly) concentrated, the product is assigned FVNL points using Option 1 (Table 2). Based on expert consultations and guidance documents [7,8], these products are assigned the highest point (8) if the only non-FVNL ingredient is ascorbic acid or similar.

Products in other food categories that contain concentrated FVNL are evaluated under Option 2—even if one of the first three ingredients is a concentrated FVNL component—because the product is not considered to be concentrated (e.g., soups containing tomato paste, granola bars with dried fruit). For packaged foods consisting of two or more products (e.g., salads with dressing packets, cracker and hummus snack kits), the average number of FVNL points for the whole item is estimated using Option 2 and rounded to the nearest point (e.g., 0, 1, 2, 5 or 8 under the FSANZ NPSC and HSR system). If an ingredient has sub-ingredients in brackets, it is counted as FVNL if one of the first two sub-ingredients is FVNL. For example, if the first ingredient were “peanut butter (peanuts, vegetable oil, salt)”, the product would score a 2 since the first ingredient in brackets is peanuts and is therefore counted as FVNL. Conversely, if the first ingredient were “peanut spread (palm oil, sugar, peanuts)”, the product would score no FVNL points since the first two ingredients in brackets are not FVNL. The FVNL points for Nutri-Score are assigned similarly to as described for the FSANZ NPSC and HSR system; however, considering the guidelines of different NP models, the highest scores (i.e., 5 or 10, depending on whether it is considered a food or beverage under Nutri-Score) are different among these models.

### 2.4. Estimation of FVNL Points for Products in FLIP 2017

The study sample excluded foods intended solely for children under 4 years of age (*n* = 229), meal replacements and nutritional supplements (*n* = 105), and cocktail drink mixers (*n* = 23). For products that were collected in multiple package sizes but had the same nutritional composition (based on the NFt and ingredients list), only one size was analyzed; however, all flavours or varieties of the product were included. The final analytic sample contained 17,337 unique packaged food and beverage products, which were then assigned FVNL points according to the FSANZ NPSC, HSR system and the Nutri-Score, using the methodology described above. The Ofcom NP model has been under review and awaiting revision since 2018 and was therefore not applied to FLIP 2017 [22]. A team of five researchers were involved in the calculation of FVNL points for different food categories for products in FLIP 2017. FVNL points were independently assigned by three reviewers to all products in a given food category, and a random 10% of the products in each category were checked by a fourth researcher. Discrepancies were resolved through discussion among team members. The distributions of FVNL points according to the FSANZ NPSC, the HSR system and the Nutri-Score (mean, standard deviation, minimum, maximum and quartiles) were examined for the overall sample and by food category. Analyses were conducted using RStudio (version 1.1.456, RStudio Inc., Boston, MA, USA).

## 3. Results and Discussion

With NP models becoming increasingly used for various public health nutrition policy objectives, it is important to ensure consistency in the application of these models and in the interpretation of the nutritional quality of comparable foods. This paper outlines novel methodology developed by the authors for assigning FVNL points to products without QUIDs for the purpose of calculating scores for NP models with FVNL components, and demonstrates the feasibility of applying the method to food supplies lacking QUIDs. 

The distribution of FVNL points for products in FLIP 2017 based on the FSANZ NPSC and the HSR system is shown in Table 3, presented for the total sample and by food category. Overall, the mean number of FVNL points assigned was 1.1, with the highest mean number of FVNL points for legumes (mean = 6.0, SD = 2.3), nuts and seeds (mean = 6.0, SD = 2.6) and vegetables (mean = 6.0, SD = 2.6). These results are unsurprising as many products in these categories tend to be FVNL-based or contain a large proportion of FVNL ingredients. In comparison, fruits and fruit juices scored fewer FVNL points, on average (mean = 4.0, SD = 3.0), perhaps at least in part due to the fact that this category includes fruit juices and drinks, many of which contain significant amounts of water and/or added sugars, thereby receiving fewer FVNL points. FVNL points were typically lowest for eggs, fats and oils, and marine and fresh water animals (i.e., seafood; mean = 0.0, SD = 0.2 for all), with most products in these categories containing no or only minimal amounts of FVNL ingredients. Previous research examining which nutrients or components are important in predicting the HSR score of a product indicated that while the number of FVNL points was not predictive of the HSR for some food categories (e.g., coffee, confectionary, dairy, beverages, desserts, pizzas), FVNL points likely play a role in driving the HSR score for FVNL-based food categories (i.e., fruits, vegetables, nuts and seeds, and legumes) [26].

Similar distributions were generated for FVNL points based on the Nutri-Score system (Supplemental Table S1), with the highest mean number of FVNL points observed among legumes (mean = 5.0, SD = 1.3), nuts and seeds (mean = 5.0, SD = 1.1) and vegetables (mean = 5.0, SD = 1.1). For certain food categories, however, the distribution of Nutri-Score FVNL points was considerably different than those of the FSANZ NPSC or HSR system. For example, no FVNL points were assigned for potatoes, sweet potatoes and yams (since tubers are not considered for FVNL under Nutri-Score); however, fats and oils received more FVNL points under the Nutri-Score than the other NP models (mean = 0.1, SD = 0.4; maximum = 5.0) given the eligibility of olive, walnut and rapeseed oils for Nutri-Score FVNL points.

In 10 of the 22 food categories, products ranged from 0 to 8 FVNL points (according to the FSANZ NPSC and HSR system): bakery products; beverages; cereals and other grain products; fruit and fruit juices; miscellaneous category; salads; sauces, gravies, dips and condiments; snacks; sugars and sweets; and vegetables (Table 3). Table 4 provides descriptions and examples of products that received 1, 2, 5 or 8 FVNL points within each TRA major food category. In such food categories with a variety of products containing differing amounts of FVNL ingredients, it can be difficult to estimate the proportion of the product that is FVNL and assign points. Differentiating between 2 and 5 FVNL points can be particularly challenging if it is unclear whether non-FVNL ingredients contribute a considerable amount to the weight of the product. In general, products in FLIP 2017 received 2 FVNL points if they contained added sugars (or sweeteners, including honey) and/or other heavier, denser ingredients such as butter, cheeses, starches or cereal-based ingredients (e.g., pasta, rice, quinoa). Conversely, products with at least the first two ingredients being FVNL and containing none of the ingredients listed above typically received ≥ 5 FVNL points. In accordance with the FSANZ NPSC, HSR system and Nutri-Score guidelines [7,8,9], FVNL points were assigned more generously for the fruit and fruit juices, vegetables, nuts and seeds, and legumes categories to account for the fact that FVNL ingredients are most prevalent in these types of products. Products in these food categories with the only non-FVNL ingredients being water, oil and/or salt were sometimes eligible to receive 5 or 8 points (under the FSANZ NPSC and HSR system), depending on the food. 

While FVNL points provide a useful and important means of giving credit to foods containing greater proportions of healthier ingredients, limitations in the current NP model methodologies may enable foods without whole FVNL ingredients and with high amounts of nutrients of public health concern to receive FVNL points and, consequently, higher NP scores. For example, under the FSANZ NPSC and HSR system, potato chips are eligible to receive between 1 and 5 FVNL points, depending on the approximate proportion of the product that constitutes potatoes. Similarly, fruit drinks can receive 1 or 2 FVNL points, despite being primarily made up of water and added sugars. With the recently announced changes to the Nutri-Score FVNL methods (e.g., the inclusion of olive, walnut and rapeseed oils) and the HSR system and Ofcom NP model currently under review, there will likely be at least minor revisions to the methods for assigning FVNL points in upcoming months or years. By showing the distribution of FVNL points in various food categories and providing examples of how different types of foods are scored, this study may be useful for policymakers in making improvements to the FVNL scoring methodologies for these NP models.

This study has several strengths and limitations. The criteria for estimating FVNL points for products without QUIDs were developed by a team of researchers experienced with NP and applying different models. These methods were modified over time as needed to reflect revisions to existing NP models and to account for newly introduced models and guidelines, and included consultation with global experts in NP. Application of this methodology to a large, highly representative database of Canadian packaged foods and beverages also demonstrates the suitability of these criteria for estimating FVNL points for a variety of products. Our results showing distributions of FVNL points, overall and within different food categories, may also be somewhat generalizable to other national food supplies given that most products in FLIP 2017 are offered by multinational food companies; however, evidence also suggests that the nutritional composition of the same products often varies between countries [27]. A limitation of this work is that our research team’s criteria for estimating FVNL points for products without QUIDs have not been formally validated. This study does, however, provide evidence of its usability by demonstrating that this methodology generates expected results when applied to the Canadian packaged food supply, with products in the fruit and fruit juices, vegetables, nuts and seeds, and legumes categories receiving the most FVNL points. Nonetheless, there is still a certain amount of ambiguity in estimating FVNL points using our methodology, given the wide variety and continuously evolving nature of products in Canada’s food supply, as well as the application-specific criteria of NP models. NP models are designed to complement and support food-based dietary guidelines in the regions or countries in which they are applied, and the information presented on FVNL may not necessarily be reflective of dietary recommendations in Canada [28]. In addition, our criteria enable less precision in calculating FVNL points than when QUIDs are available, particularly for the HSR system, which involves estimating FVNL points based on a very narrow gradation of percentages of FVNL ingredients, ranging from 1 to 8 points in 1-point increments. Lastly, estimating FVNL points in the absence of QUIDs can be time-consuming and resource-intensive; our application of these methods to FLIP 2017 involved a team of five experts in NP and required several months to complete. Mandatory QUIDs in Canada should therefore be an important policy objective to ensure that NP models with FVNL components can be applied objectively, consistently and relatively easily. With NP models becoming increasingly utilized by policymakers [5], QUIDs are critical not only to the implementation of NP models, but also in evaluating and monitoring new public health nutrition policies, such as the most recently released Canada’s Food Guide, which emphasizes FVNL foods and beverages [28].

## 4. Conclusions

In summary, this study describes methodology for estimating FVNL points for products without QUIDs for the purpose of applying NP models with FVNL components. It also demonstrates the feasibility of applying these methods to packaged food supplies without QUIDs, such as in Canada. This paper may improve the objectivity, consistency and accuracy of estimating FVNL points for products without QUIDs, which is important given the proliferation of NP in public health nutrition research and policymaking. Moreover, by illustrating the distribution of FVNL points in different food categories, this study may prove useful to academic experts and regulators who are currently reviewing and revising existing NP models.

## Figures and Tables

**Table 1:** The point systems for estimating the proportion of a packaged food or beverage product containing fruits, vegetables, nuts, legumes or rapeseed, walnut and olive oils (FVNL) ^a^.

Points	Percentage of Product Containing FVNL ^a^						
	Ofcom	FSANZ NPSC ^b^		Health Star Rating System		Nutri-Score	
	Concentrated or Non-Concentrated (%) ^c^	Concentrated (%) ^d^	Non-Concentrated (%) ^e^	Concentrated (%) ^d^	Non-Concentrated (%) ^e^	Foods (%) ^f^	Beverages (%) ^g^
0	≤ 40	< 25	≤ 40	< 25	≤ 40	≤ 40	≤ 40
1	> 40	≥ 25	> 40	≥ 25	> 40	> 40	-
2	> 60	≥ 43	> 60	≥ 43	> 60	> 60	> 40
3	-	-	-	≥ 52	> 67	-	-
4	-	-	-	≥ 63	> 75	-	> 60
5	> 80	≥ 67	> 80	≥ 67	> 80	> 80	-
6	-	-	-	≥ 80	> 90	-	-
7	-	-	-	≥ 90	> 95	-	-
8	-	= 100	= 100	= 100	= 100	-	-
9	-	-	-	-	-	-	-
10	-	-	-	-	-	-	> 80

^a^ Only the Nutri-Score assigns points for rapeseed, walnut and olive oils; oils are not counted under the other nutrient profile models listed in the table. ^b^ FSANZ NPSC: Food Standards Australia New Zealand Nutrient Profiling Scoring Criterion. ^c^ The Ofcom NP model FVNL point system does not distinguish between concentrated and non-concentrated foods and beverages in terms of the percentage of the product containing FVNL. ^d^ The FVNL point system for concentrated foods and beverages (i.e., a concentrated FVNL component must be one of the first three ingredients listed), such as fruit juice concentrate. ^e^ The FVNL point system for non-concentrated foods and beverages (i.e., a concentrated FVNL component is not one of the first three ingredients listed). ^f^ The Nutri-Score FVNL point system for foods, which does not apply to: mineral water and spring water; flavoured water; fruit juices, nectars and smoothies; vegetable juices; drinks with added sugar and/or sweeteners; teas, infusions or coffee reconstituted exclusively with water. ^g^ The Nutri-Score FVNL point system for beverages, which applies to: mineral water and spring water; flavoured water; fruit juices, nectars and smoothies; vegetable juices; drinks with added sugar and/or sweeteners; teas, infusions or coffee reconstituted exclusively with water.

**Table 2:** Overview of the criteria used for estimating the contents of fruit, vegetable, nut and legume (FVNL) of products according to the Food Standards Australia New Zealand Nutrient Profiling Scoring Criterion (FSANZ NPSC) and the Health Star Rating (HSR) system ^a^.

Option 1 (Concentrated) ^b^			Option 2 (Non-Concentrated)		
Points ^c^	% Concentrated	Criteria	Points ^c^	% Non-Concentrated	Criteria
0	< 25	Concentrated FVNL is not one of the first three ingredients	0	≤ 40	FVNL is not one of the first two ingredients
1	≥ 25	Concentrated FVNL is the third ingredient	1	> 40	FVNL is the second ingredient
2	≥ 43	Concentrated FVNL is the first or second ingredient, but non-FVNL ingredients appear to substantially contribute to the product’s weight (e.g., Concentrated apple juice, water, sugar)	2	> 60	FVNL is the first ingredient, but non-FVNL ingredient(s) appear to substantially contribute to the product’’s weight (e.g., Cherries, water, sugar)
5	≥ 67	FVNL is the first ingredient, and non-FVNL ingredients are not considered to contribute a substantial amount to the product’s weight (e.g., Thompson raisins, sunflower oil)	5	> 80	FVNL is the first ingredient, and non-FVNL ingredients are not considered to contribute a substantial amount to the product’s weight (e.g.: Cherries, safflower oil, vinegar (as a coating); Almonds, salt, preservatives, etc.); if other ingredients appear after salt or preservatives, then this ingredient is considered to contribute only a small amount
8	= 100	Concentrated FVNL is the only ingredient(s) contributing to the product’s weight; other non-FVNL ingredient(s) listed that contribute only minimally to the product’s weight may be: salt, preservatives, colour, vitamins, minerals, oil, flavour extracts, antioxidants, food additives; if other ingredients appear after salt or preservatives, this ingredient is considered to contribute only a small amount; E.g., Apple and/or pear concentrate, apple and/or grape and/or pear juice concentrate, citrus pectin, elderberry juice concentrate, natural flavour, lemon juice concentrate	8	= 100	FVNL is the only ingredient(s) contributing to the product’s weight, in a "pure state" (e.g., raw apples, no added sugar); other non-FVNL ingredient(s) listed that contribute only minimally to the product’s weight may be: preservatives (salt if it is clearly a preservative), colour, vitamins, minerals, flavour extracts, antioxidants, food additives; E.g., Almonds, wheat starch, citric acid; E.g., Beets, water, salt ^d^

^a^ FVNL points for the Nutri-Score are assigned similarly to as described for the FSANZ NPSC and the HSR system; however, the highest scores (i.e., 5 or 10, depending on whether it is considered a food or beverage under Nutri-Score) are different between these models and are not shown in this table. Criteria specific to estimating FVNL points when applying the Ofcom nutrient profile model have been published elsewhere: [21]. ^b^ If the product is a fruit or vegetable (including fruit and vegetable juices or drinks) containing FVNL components that are all (or mainly) concentrated, the product is assigned FVNL points using Option 1; products in all other categories with one or more concentrated ingredients (e.g., soups with tomato paste, trail mixes with dried fruit, soft drinks containing concentrated fruit juice) are assessed under Option 2. ^c^ For the HSR system, FVNL points actually range from 1 to 8 points in 1-point increments [8], based on additional percentages not listed here; however, it is not possible to achieve this level of specificity in the absence of quantitative ingredient declarations. ^d^ Canned vegetables packed in water with no additional non-FVNL ingredients (except salt as a preservative) receive 8 FVNL points as this water is typically drained prior to consumption; this is consistent with the guidelines for the FSANZ NPSC and the HSR system [7,8].

**Table 3:** Distribution of fruit, vegetable, nut and legume (FVNL) points in Canadian packaged foods and beverages, as defined by the Food Standards Australia New Zealand Nutrient Profiling Scoring Criterion and the Health Star Rating system, presented for the total sample and by food category ^a^.

Food Category ^a^	Number of Products	Distribution of FVNL Points					
		Mean (SD)	Min	25 th	50 th	75 th	Max
TOTAL	17,337	1.1 (2.2)	0	0	0	1	8
A. Bakery products	2775	0.2 (0.8)	0	0	0	0	8
B. Beverages	852	0.3 (1.2)	0	0	0	0	8
C. Cereals and other grain products	1276	0.3 (1.2)	0	0	0	0	8
D. Dairy products	1498	0.1 (0.3)	0	0	0	0	2
E. Desserts	679	0.1 (0.3)	0	0	0	0	2
F. Dessert toppings and fillings	94	0.5 (0.9)	0	0	0	2	2
G. Eggs and egg substitutes	61	0.0 (0.2)	0	0	0	0	1
H. Fats and oils	656	0.0 (0.2)	0	0	0	0	2
I. Marine and fresh water animals	446	0.0 (0.2)	0	0	0	0	2
J. Fruit and fruit juices	1061	4.0 (3.0)	0	2	2	8	8
K. Legumes	188	6.0 (2.3)	1	5	5	8	8
L. Meat, poultry, their products and substitutes	962	0.1 (0.4)	0	0	0	0	5
M. Miscellaneous	558	0.9 (1.8)	0	0	0	1	8
N. Combination dishes	1139	0.4 (0.7)	0	0	0	1	5
O. Nuts and seeds	255	6.0 (2.6)	1	5	8	8	8
P. Potatoes, sweet potatoes and yams	132	4.0 (2.3)	2	2	2	5	8
Q. Salads	130	3.0 (2.1)	0	1	2	5	8
R. Sauces, dips, gravies and condiments	1250	1.0 (1.5)	0	0	1	2	8
S. Snacks	865	2.0 (2.0)	0	0	2	2	8
T. Soups	480	0.5 (0.6)	0	0	0	1	5
U. Sugars and sweets	1109	0.7 (1.4)	0	0	0	1	8
V. Vegetables	871	6.0 (2.6)	0	5	8	8	8

^a^ Food categories are defined in Health Canada’s Table of Reference Amounts for Food [25].

**Table 4:** Examples of types of Canadian packaged foods and beverages in each food category ^a^ that received 1, 2, 5 or 8 fruit, vegetable, nut and legume (FVNL) points, as defined by the Food Standards Australia New Zealand Nutrient Profiling Scoring Criterion and the Health Star Rating system.

Food Category ^a^	FVNL Points			
	1	2	5	8
A. Bakery products	Crackers, cereal bars, fruit pies and cakes with nuts or dried fruit as the second ingredient	Crackers, cereal bars, fruit pies and cakes with nuts, or fresh or dried fruit as the first ingredient	Fruit and nut bars, vegetable-based crackers, and vegetable- or seed-based wraps with FVNL as the first two or more ingredients	Fruit and nut bars with no added sugar or fat
B. Beverages	Fruit drinks, energy drinks, sodas or flavoured waters with concentrated or non-concentrated fruit as the second ingredient	Coconut water with added sugars; soft drinks with concentrated or non-concentrated fruit as the first ingredient and containing added sugars	Flavoured water with concentrated or non-concentrated fruit as the first ingredient and no added sugars	Coconut water with no added sugars
C. Cereals and other grain products	Breakfast cereals with dried fruit as the second ingredient; flavoured pastas and rice dishes with vegetables as the second ingredient	Legume-based breakfast cereals with brown rice and/or added sugars; gnocchi with potatoes as the first ingredient	Breakfast cereals with FVNL as the first ingredient with no added sugars (but containing other non-FVNL ingredients)	Plain seeds (e.g., chia, hemp, flax)
D. Dairy products	Cheeses, yogurts or plant-based milk beverages with fruit or nuts as the second ingredient	Yogurts or non-dairy cheeses with fruit or nuts as the first ingredient and containing added sugars and/or significant amounts of non-FVNL ingredients ^c^	N/A ^b^	N/A ^b^
E. Desserts	Ice creams, frozen yogurts or fruit-based desserts (e.g., peaches in peach gel) with fruit or nuts as the second ingredient	Fruit ice bars with fruit as the first ingredient and containing added sugars	N/A ^b^	N/A ^b^
F. Dessert toppings and fillings	Pie fillings with dried fruit as the second ingredient	Pie fillings with fruit as the first ingredient and containing added sugars and significant amounts of other non-FVNL ingredients ^c^	N/A ^b^	N/A ^b^
G. Eggs and egg substitutes	Liquid egg mixtures with vegetables as the second ingredient	N/A^b^	N/A ^b^	N/A ^b^
H. Fats and oils	Olive oils or salad dressings with vegetables or fruits as the second ingredient	Salad dressings with vegetables or fruits as the first ingredient, and containing considerable amounts of non-FVNL ingredients (e.g., oils, vinegars, added sugars) ^c^	N/A ^b^	N/A ^b^
I. Marine and fresh water animals	Mussels, salmon burgers or herring with vegetables as the second ingredient	Anchovy fish pastes with vegetables as the first ingredient	N/A ^b^	N/A ^b^
J. Fruit and fruit juices	Non-concentrated fruit packaged in water or syrup, with fruit as the second ingredient; concentrated fruit juices or drinks with fruit as the third ingredient (e.g., "Filtered water, sugar, concentrated fruit juice”); smoothies with fruit puree as the second ingredient	Fruit packed in water or syrup with fruit as the first ingredient and containing added sugars; fruit juices or cocktails with concentrated fruit juice as the second ingredient (with or without added sugars)	Fruit packed in water with fruit as the first ingredient and containing no added sugars; concentrated or non-concentrated fruit juices and drinks with fruit as the first ingredient and containing water but no added sugars	Plain frozen or dried fruits, fruit juices and canned fruits packed in fruit juice with no added sugars or water
K. Legumes	Tofu-based products with soybeans as the second ingredient	Tempeh with soybeans as the first ingredient; soup mix with beans as the first ingredient and containing considerable amounts of non-FVNL ingredients (e.g., barley) ^c^	Canned legumes with legumes as the first ingredient and containing water and salt	Dried or canned legumes without added salt
L. Meat, poultry, their products and substitutes	Turkey, chicken, sausage or plant-based meat alternatives with vegetables or legumes as the second ingredient	Plant-based meat alternatives with vegetables or legumes as the first ingredient containing water, oils, rice or other non-FVNL ingredients as one of the first three ingredients	Plant-based meat alternatives with vegetables or legumes as the first ingredient, with the first three ingredients all FVNL or non-FVNL ingredients not considered to contribute a substantial amount to the product (e.g., sodium bicarbonate)	N/A ^b^
M. Miscellaneous	Seasonings with dehydrated vegetables or spices as the second ingredient	Sweetened shredded, flaked or desiccated coconut with coconut as the first ingredient and containing added sugars; seasonings with dehydrated vegetables or spices as the first ingredient and containing added sugars and/or considerable amounts of other non-FVNL ingredients ^c^	Seasonings with dehydrated vegetables or spices as the first ingredient, containing primarily FVNL ingredients (and no added sugars), with smaller amounts of oil or other non-FVNL ingredients ^c^	Unsweetened shredded, flaked or desiccated coconut; seasonings containing only vegetables or spices
N. Combination dishes	Ready-meals or side dishes with vegetables, nuts or legumes as the second ingredient	Ready-meals or side dishes with vegetables, nuts or legumes as the first ingredient but containing substantial amounts of non-FVNL ingredients (e.g., meat, cheese, starches, grains, water) ^c^	Ready-meals or side dishes with vegetables, nuts or legumes as the first ingredient and smaller amounts of non-FVNL ingredients (e.g., sweet potato noodles) ^c^	N/A ^b^
O. Nuts and seeds	Pastes or doughs with nuts as the second ingredient (e.g., almond candy dough)	Nut butters with nuts as the first ingredient but containing substantial amounts of non-FVNL ingredients (e.g., peanut butter with added sugars) ^c^	Nut butters with nuts as the first ingredient and containing oil and/or salt as the only other ingredients ^c^	Plain nuts; nut butters containing only nuts with no additional ingredients
P. Potatoes, sweet potatoes and yams	N/A	Frozen or prepared potato dishes with potatoes as the first ingredient and containing substantial amounts of other ingredients (e.g., oils, cheeses, starches) ^c^	Frozen or prepared potato dishes with potatoes as the first ingredient and containing smaller amounts of non-FVNL ingredients (e.g., oils, salt) ^c^	Plain raw potatoes or canned potatoes containing only water and salt
Q. Salads	Chicken-, tuna- or grain-based salads with vegetables or legumes as the second ingredient	Caesar and potato salad mixes or coleslaw with FVNL as the first ingredient but containing considerable amounts of non-FVNL ingredients (e.g., meat, cheese, pasta or grains) ^c^	Vegetable- or bean-based salad mixes with FVNL as the first ingredient and containing smaller amounts of non-FVNL ingredients (e.g., spinach, kale or Greek salads)	Salad mix containing only greens (e.g., chard, spinach, arugula)
R. Sauces, dips, gravies and condiments	Sauces with vegetables, nuts or legumes as the second ingredient; balsamic vinegars with grape must as the second ingredient; mustard with mustard seeds as the second ingredient	Pasta sauces, salsas, hummus and other condiments with vegetables, nuts or legumes as the first ingredient and containing considerable amounts of non-FVNL ingredients ^c^	Hummus with chickpeas and tahini as the first two ingredients; pasta sauces and salsas with tomatoes as the first ingredient and smaller amounts of non-FVNL ingredients ^c^	Salsas, dips and other condiments with only FVNL ingredients ^c^
S. Snacks	Sweet or savoury snacks with FVNL as the second ingredient (e.g., corn chips with flaxseed)	Sweet or savoury snacks with FVNL as the first ingredient and significant amounts of non-FVNL ingredients (e.g., potato chips with non-FVNL seasonings, sugar-coated nuts) ^c^	Sweet or savoury snacks with FVNL as the first ingredient and smaller amounts of non-FVNL ingredients (e.g., potato chips or nuts containing only oil and/or salt) ^c^	Sweet or savoury snacks containing only FVNL ingredients (e.g., apple chips; trail mixes with only plain nuts, seeds and dried fruits)
T. Soups	Water- or broth-based soups with vegetables or legumes as the second ingredient	Soups with vegetables or legumes as the first ingredient and containing considerable amounts of non-FVNL ingredients (e.g., tomato, onion or butternut squash soups)	Soups with vegetables or legumes as the first ingredient and smaller amounts of non-FVNL ingredients (e.g., vegetable- or bean-based soups) ^c^	N/A ^b^
U. Sugars and sweets	Confectionary, jams, jellies or spreads with fruit or nuts as the second ingredient (e.g., milk chocolate with nuts, strawberry jelly)	Confectionary, jams, jellies or spreads with fruit or nuts as the first ingredient and containing substantial amounts of non-FVNL ingredients (e.g., fruit leather, fruit spreads or jams with added sugars) ^c^	Fruit snacks and spreads with fruit as the first ingredient and containing smaller amounts of non-FVNL ingredients (e.g., fruit leather or spreads without added sugars but containing other non-FVNL ingredients) ^c^	Fruit leather or spreads with only FVNL ingredients
V. Vegetables	Canned vegetables and vegetable juices or cocktails with vegetables as the second ingredient; concentrated vegetable juices or drinks with a vegetable as the third ingredient	Canned or pickled vegetables or vegetable pastes with vegetables as the first ingredient and containing sugars, corn starch and/or considerable amounts of other non-FVNL ingredients; concentrated vegetable juices or drinks with vegetables as the second ingredient	Products with vegetables as the first ingredient but containing smaller amounts of non-FVNL ingredients (e.g., vinegar, wine, oil)	Plain frozen vegetables; canned vegetables packaged in tomato juice, water and/or salt; vegetable juices containing only vegetables

^a^ Food categories are defined in Health Canada’s Table of Reference Amounts for Food [25]. ^b^ No products in that food category received that number of FVNL points. ^c^ “Amounts” refer to the placement of products within a product’s ingredients list (e.g., “smaller” amounts refer to ingredients located near the end of the ingredients list or that are fewer in number, while “considerable” or “significant” amounts describe ingredients featured closer to the beginning of the ingredients list or that are greater in number).

## Supplementary Materials

The following are available online at https://www.mdpi.com/2304-8158/9/8/1127/s1, Table S1: Distribution of fruit, vegetable, nut, legume and olive oil, walnut oil and rapeseed oil (FVNL) points as defined by the Nutri-Score system, presented for the total sample and by food category.

## References

[1] World Health Organization Nutrient Profiling. https://www.who.int/nutrition/topics/profiling/en/.

[2] United States Department of Agriculture Special Supplemental Nutrition Program for Women, Infants and Children (WIC): Revisions in the WIC Food Packages. https://www.federalregister.gov/documents/2014/03/04/2014-04105/special-supplemental-nutrition-program-for-women-infants-and-children-wic-revisions-in-the-wic-food.

[3] The National Food Agency of Sweden Regulations amending the National Food Agency’s Regulations (SLVFS 2005:9) on the Use of a Particular Symbol. https://www.livsmedelsverket.se/globalassets/om-oss/lagstiftning/livsmedelsinfo-till-konsum---markning/livsfs-2015-1-particular-symbol-eng.pdf?AspxAutoDetectCookieSupport=1.

[4] World Health Organization Set of Recommendations on the Marketing of Foods and Non-alcoholic Beverages to Children. http://www.who.int/dietphysicalactivity/publications/recsmarketing/en/.

[5] Labonté M., Poon T., Gladanac B., Ahmed M., Franco-Arellano B., Rayner M., L’Abbé M.R. (2018). Nutrient Profile Models with Applications in Government-Led Nutrition Policies Aimed at Health Promotion and Noncommunicable Disease Prevention: A Systematic Review. Adv. Nutr..

[6] UK Department of Health Nutrient Profiling Technical Guidance. https://www.gov.uk/government/publications/the-nutrient-profiling-model.

[7] Food Standards Australia New Zealand Short Guide for Industry to the Nutrient Profiling Scoring Criterion in Standard 1.2.7—Nutrition, Health and Related Claims. https://www.foodstandards.gov.au/industry/labelling/Documents/Short-guide-for-industry-to-the-NPSC.pdf.

[8] Guide for Industry to the Health Star Rating Calculator (HSRC). http://healthstarrating.gov.au/internet/healthstarrating/publishing.nsf/content/E380CCCA07E1E42FCA257DA500196044/$File/Guide%20for%20Industry%20to%20the%20Health%20Star%20Rating%20Calculator.pdf.

[9] Nutri-Score Frequently Asked Questions. www.santepubliquefrance.fr/QR_scientifique_technique_EN_011119.

[10] Food Standards Australia New Zealand Overview of the Nutrient Profiling Scoring Criterion. http://www.foodstandards.gov.au/industry/labelling/Pages/Consumer-guide-to-NPSC.aspx.

[11] Australian Government About Health Star Ratings. http://www.healthstarrating.gov.au/internet/healthstarrating/publishing.nsf/Content/About-health-stars.

[12] Santé Publique France Nutri-Score. https://www.santepubliquefrance.fr/determinants-de-sante/nutrition-et-activite-physique/articles/nutri-score.

[13] Dréano-Trécant L., Egnell M., Hercberg S., Galan P., Soudon J., Fialon M., Touvier M., Kesse-Guyot E., Julia C. (2020). Performance of the Front-of-Pack Nutrition Label Nutri-Score to Discriminate the Nutritional Quality of Foods Products: A Comparative Study across 8 European Countries. Nutrients.

[14] European Union Commission Notice on the Application of the Principle of Quantitative Ingredients Declaration (QUID). https://www.fsai.ie/uploadedFiles/CommissionNoticeC393_05_QUID.pdf.

[15] Government of the United Kingdom Food Labelling: Giving Food Information to Consumers. https://www.gov.uk/guidance/food-labelling-giving-food-information-to-consumers.

[16] Food Standards Australia New Zealand Ingredient Labelling of Foods User Guide to Standard 1.2.4—Labelling of Ingredients. https://www.foodstandards.gov.au/code/userguide/Documents/Guide%20to%20Standard%201.2.4%20-%20Ingredient%20Labelling%20of%20Foods.pdf.

[17] Rayner M., Wood A., Lawrence M., Mhurchu C.N., Albert J., Barquera S., Friel S., Hawkes C., Kelly B., Kumanyika S. (2013). Monitoring the health-related labelling of foods and non-alcoholic beverages in retail settings. Obes. Rev..

[18] Government of Canada Food Label: Ingredient List. https://www.canada.ca/en/health-canada/services/understanding-food-labels/ingredient-list.html.

[19] Franco-Arellano B., Kim M.A., Vandevijvere S., Bernstein J.T., Labonté M., Mulligan C., L’Abbé M.R. (2019). Assessment of Packaged Foods and Beverages Carrying Nutrition Marketing against Canada’s Food Guide Recommendations. Nutrients.

[20] Bernstein J.T., Franco-Arellano B., Schermel A., Labonté M., L’Abbé M.R. (2017). Healthfulness and nutritional composition of Canadian prepackaged foods with and without sugar claims. Appl. Physiol. Nutr. Metab..

[21] Poon T., Labonté M., Mulligan C., Ahmed M., Dickinson K.M., L’Abbé M.R. (2018). Comparison of nutrient profiling models for assessing the nutritional quality of foods: A validation study. Br. J. Nutr..

[22] Public Health England UK Nutrient Profiling Model 2018 review. https://www.gov.uk/government/consultations/consultation-on-the-uk-nutrient-profiling-model-2018-review.

[23] Australian Government Formal review of the System after Five Years of Implementation (June 2014 to June 2019). http://www.healthstarrating.gov.au/internet/healthstarrating/publishing.nsf/Content/formal-review-of-the-system-after-five-years.

[24] Franco-Arellano B., Arcand J., Kim M.A., Schermel A., L’Abbé M. (2020). Progress towards reducing industrially-produced trans-fatty acids in the Canadian marketplace, 2013-2017. Public Health Nutr..

[25] Canadian Food Inspection Agency Reference Amounts. https://www.inspection.gc.ca/food/requirements-and-guidance/labelling/-f-for-industry/-f-nutrition-labelling/-f-nutrition-facts-table/eng/1502483894184/1502483895254?chap=5.

[26] Ahmed M., Orenga A., Lou W., Green H., L’Abbé M. Which nutrient(s) are important in predicting the scoring of a food product by the Health Star Rating system? A machine learning approach.

[27] Access to Nutrition Foundation Access to Nutrition Index: Global Index 2018. https://accesstonutrition.org/index/global-index-2018/.

[28] Government of Canada Canada’s Food Guide. https://food-guide.canada.ca/en/.

