# Supplementary material for: Methodology for the Determination of Fruit, Vegetable, Nut and Legume Points for Food Supplies without Quantitative Ingredient Declarations and Its Application to a Large Canadian Packaged Food and Beverage Database
Source: Foods. 2020 Aug 15;9(8):1127. doi: 10.3390/foods9081127 (PMC7465889; doi:10.3390/foods9081127)
Supplement: Supplementary file 1 [file foods-09-01127-s001.pdf]

SUPPLEMENTARY MATERIAL

Methodology for the Determination of Fruit, Vegetable, Nut and Legume Points for Food Supplies without Quantitative Ingredient Declarations and its Application to a Large Canadian Packaged Food and Beverage Database

Laura Vergeer [1], Mavra Ahmed [1], Beatriz Franco-Arellano [1], Christine Mulligan [1], Kacie Dickinson [1,2], Jodi T. Bernstein [1], Marie-Ève Labonté [1,3] and Mary R. L'Abbé [1,*]

[1] Department of Nutritional Sciences, Faculty of Medicine, University of Toronto, Toronto, ON M5S 1A8, Canada; laura.vergeer@mail.utoronto.ca (L.V.); mavz.ahmed@utoronto.ca (M.A.); beatriz.francoarellano@mail.utoronto.ca (B.F.A.); christine.mulligan@mail.utoronto.ca (C.M.); jodi.bernstein@mail.utoronto.ca (J.T.B.)
[2] Caring Futures Institute, College of Nursing and Health Sciences, Flinders University, Adelaide, SA 5001, Australia; kacie.dickinson@flinders.edu.au (K.D.)
[3] Centre Nutrition, Santé et Société (NUTRISS), Institute of Nutrition and Functional Foods, Laval University, Québec City, QC G1V 0A6, Canada; marie-eve.labonte@fsaa.ulaval.ca (M.E.L.)
* Correspondence: mary.labbe@utoronto.ca (M.R.L.); Tel.: +1 416-946-7545

Table S1. Distribution of fruit, vegetable, nut, legume and olive oil, walnut oil and rapeseed oil (FVNL) points as defined by the Nutri-Score system, presented for the total sample and by food category[a].

Food category[a]	Number of products	Mean (SD)	Distribution of FVNL points				
			Min	25th	50th	75th	Max
TOTAL	17,337	1.0 (2.0)	0	0	0	1	10
A. Bakery products	2,775	0.2 (0.7)	0	0	0	0	5
B. Beverages	852	0.4 (1.5)	0	0	0	0	10
C. Cereals and other grain products	1,276	0.2 (0.9)	0	0	0	0	5
D. Dairy products	1,498	0.1 (0.4)	0	0	0	0	5
E. Desserts	679	0.1 (0.5)	0	0	0	0	2
F. Dessert toppings and fillings	94	0.5 (0.9)	0	0	0	2	2
G. Eggs and egg substitutes	61	0.0 (0.2)	0	0	0	0	1
H. Fats and oils	656	0.1 (0.4)	0	0	0	0	5
I. Marine and fresh water animals	446	0.0 (0.2)	0	0	0	0	2
J. Fruit and fruit juices	1,061	4.0 (2.8)	0	2	4	5	10
K. Legumes	188	5.0 (1.3)	1	5	5	5	5
L. Meat, poultry, their products and substitutes	962	0.1 (0.4)	0	0	0	0	5
M. Miscellaneous	558	0.1 (0.7)	0	0	0	0	5
N. Combination dishes	1,139	0.4 (0.7)	0	0	0	1	5
O. Nuts and seeds	255	5.0 (1.1)	0	5	5	5	5
P. Potatoes, sweet potatoes and yams	132	0.0 (0.0)	0	0	0	0	0
Q. Salads	130	3.0 (2.2)	0	0	5	5	5
R. Sauces, dips, gravies and condiments	1,250	1.0 (1.4)	0	0	1	2	5
S. Snacks	865	2.0 (2.1)	0	0	0	5	5
T. Soups	480	0.6 (0.9)	0	0	0	1	5
U. Sugars and sweets	1,109	0.7 (1.2)	0	0	0	1	5
V. Vegetables	871	5.0 (1.1)	0	5	5	5	10

[a]Food categories are defined in Health Canada's Table of Reference Amounts for Food [26].
